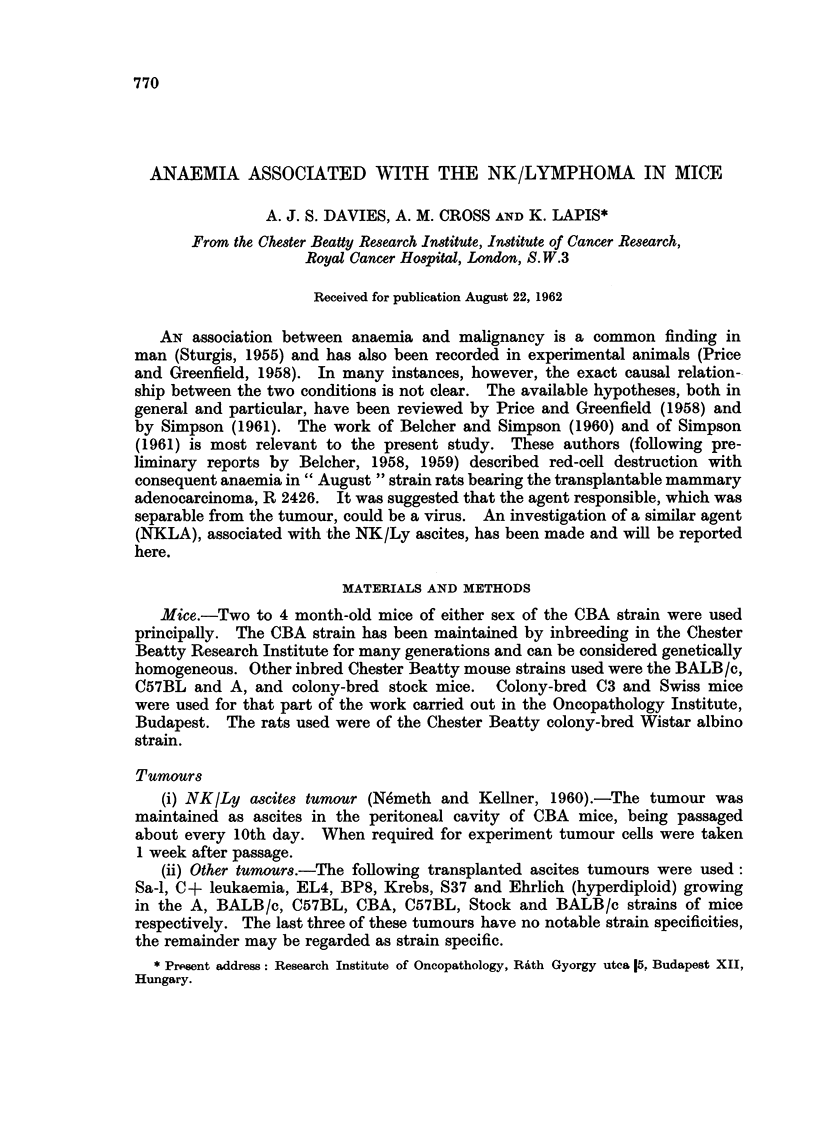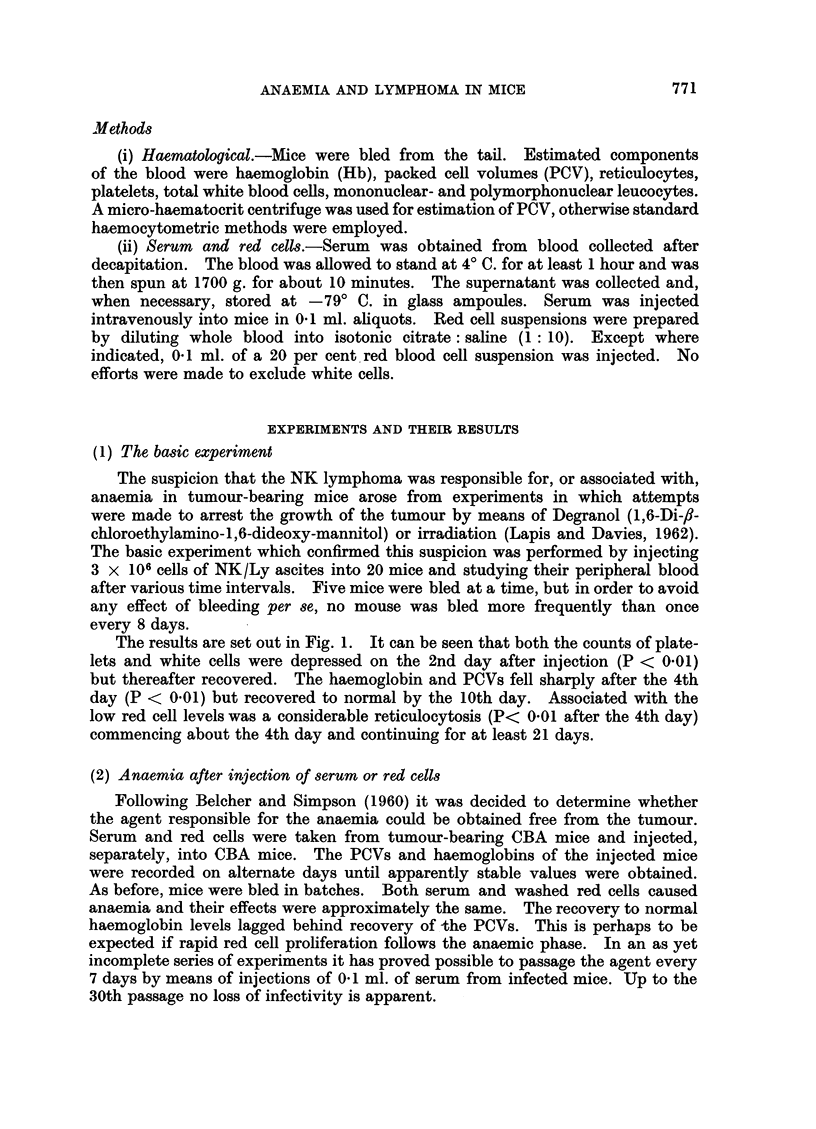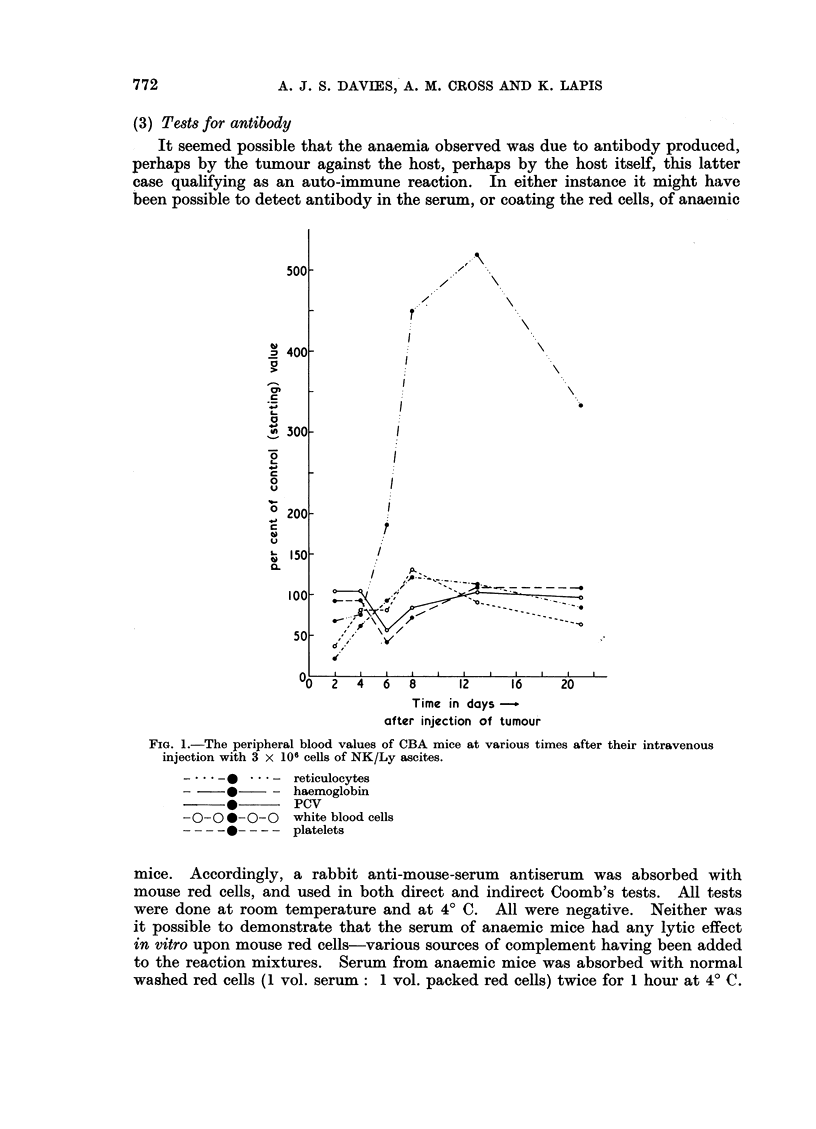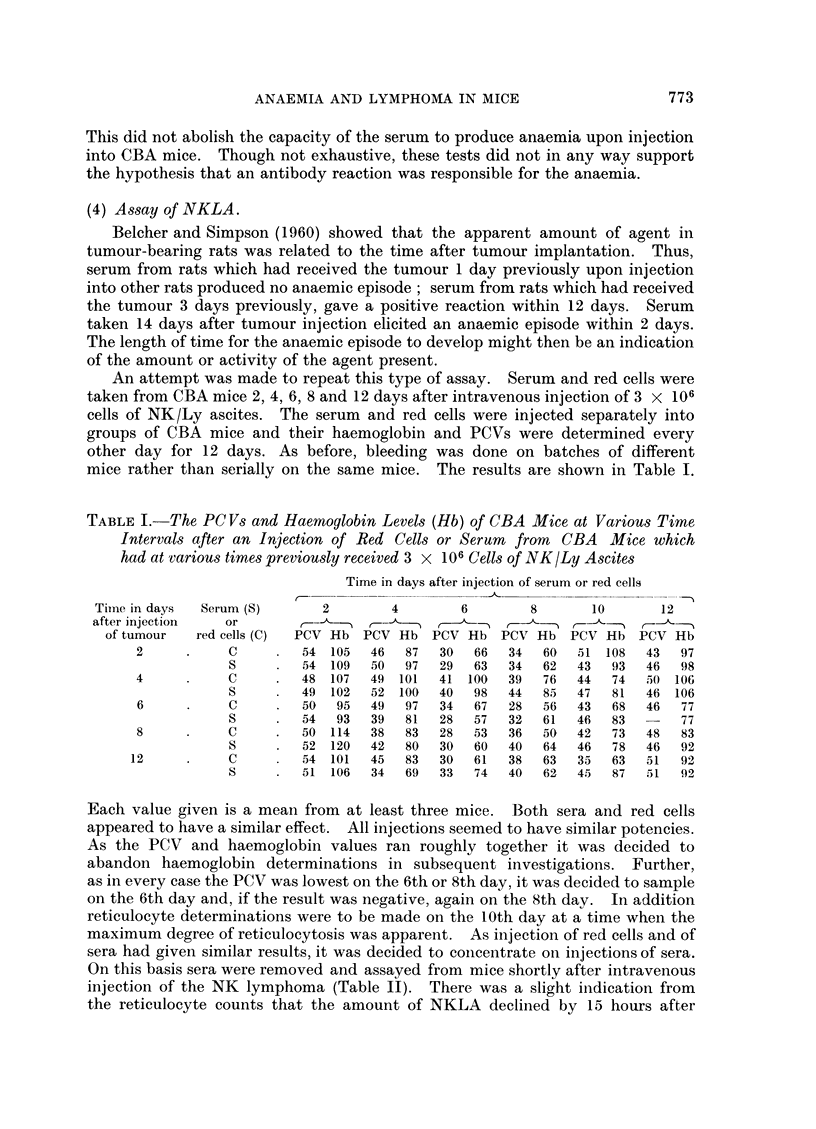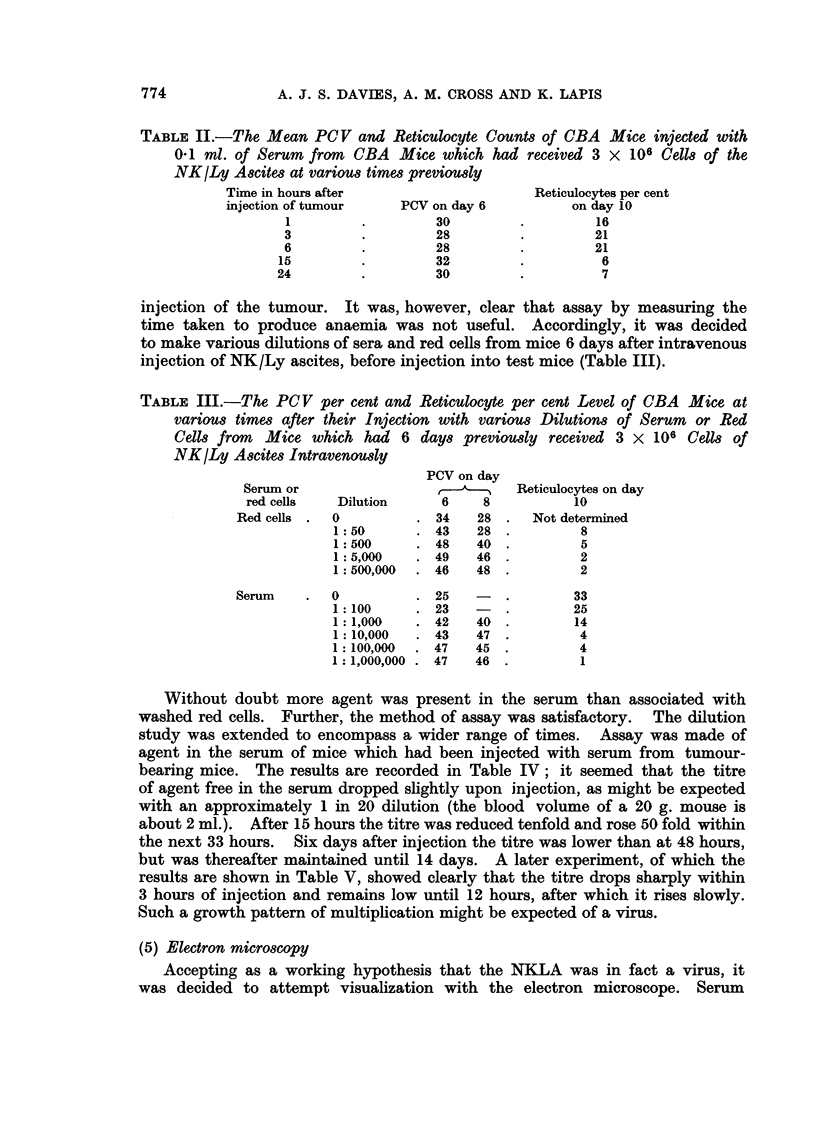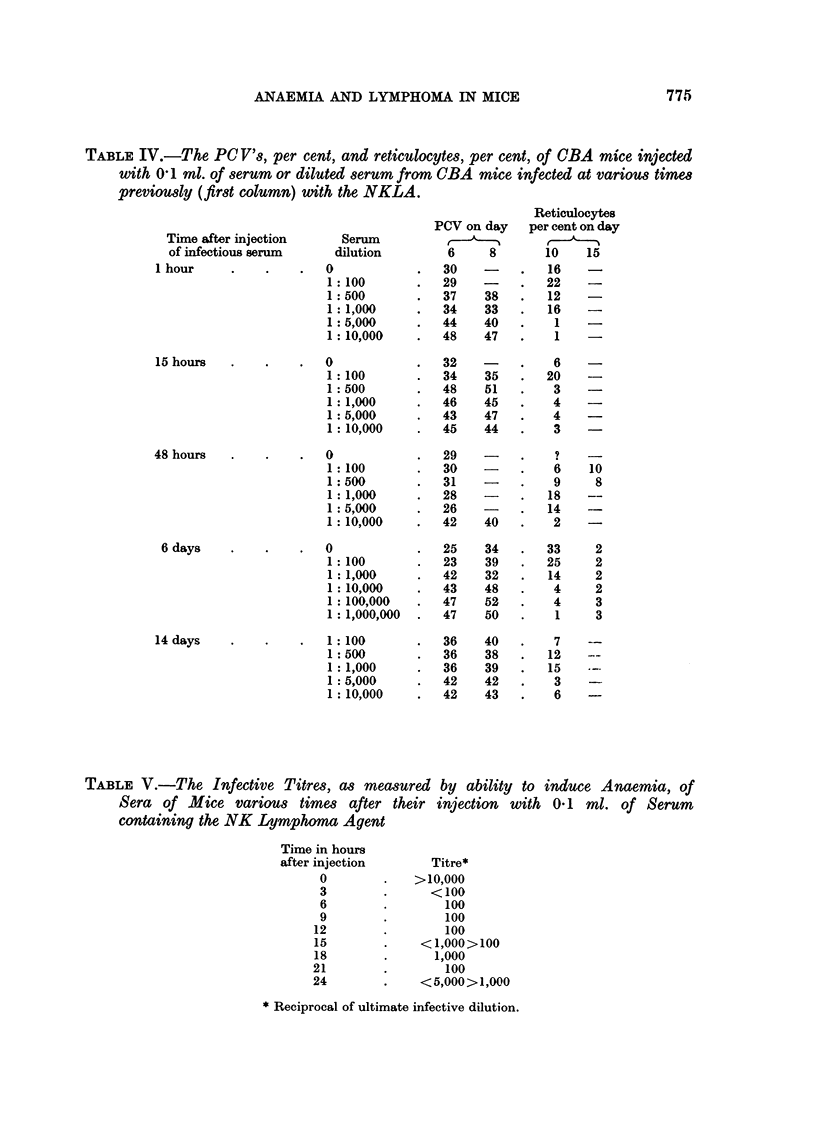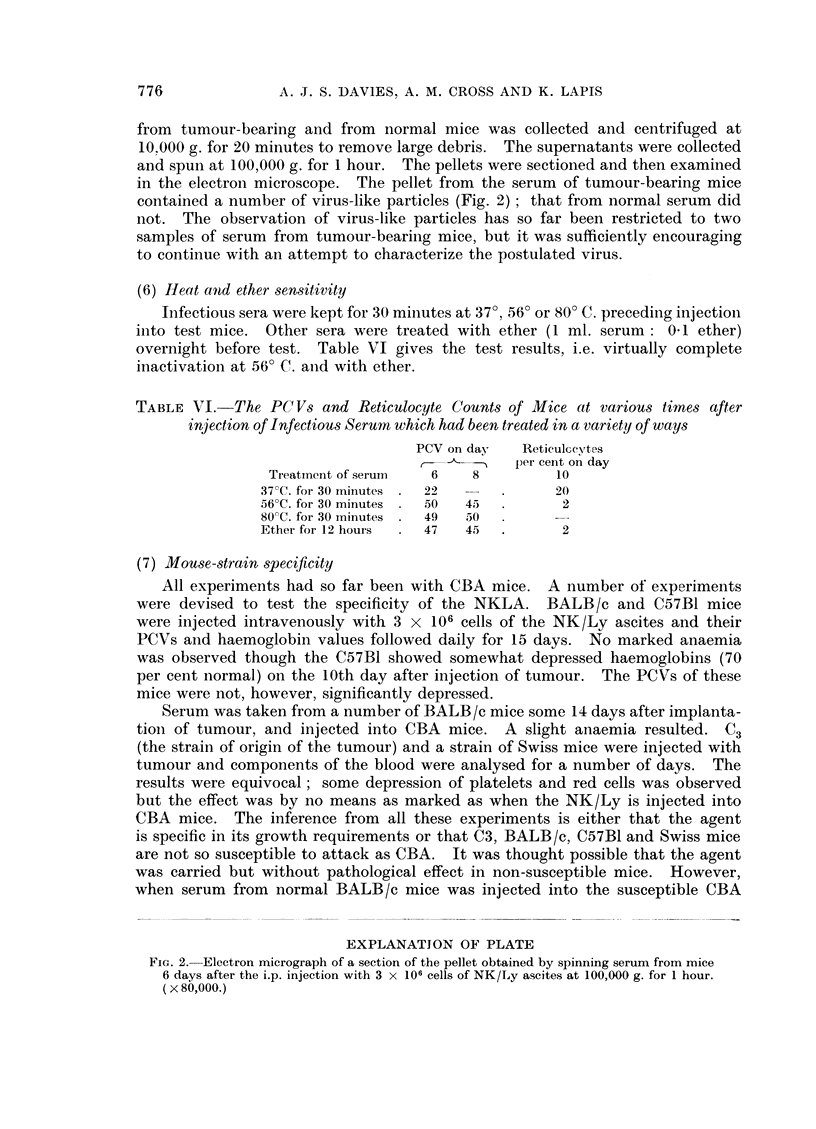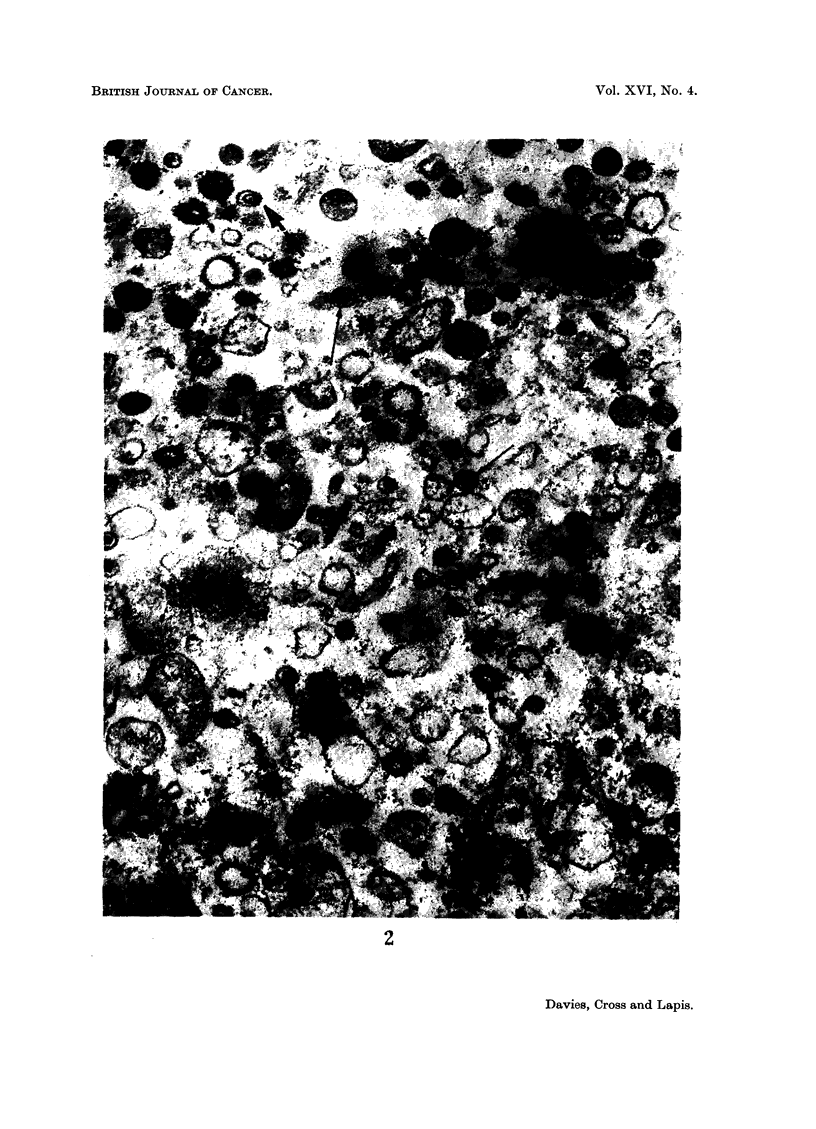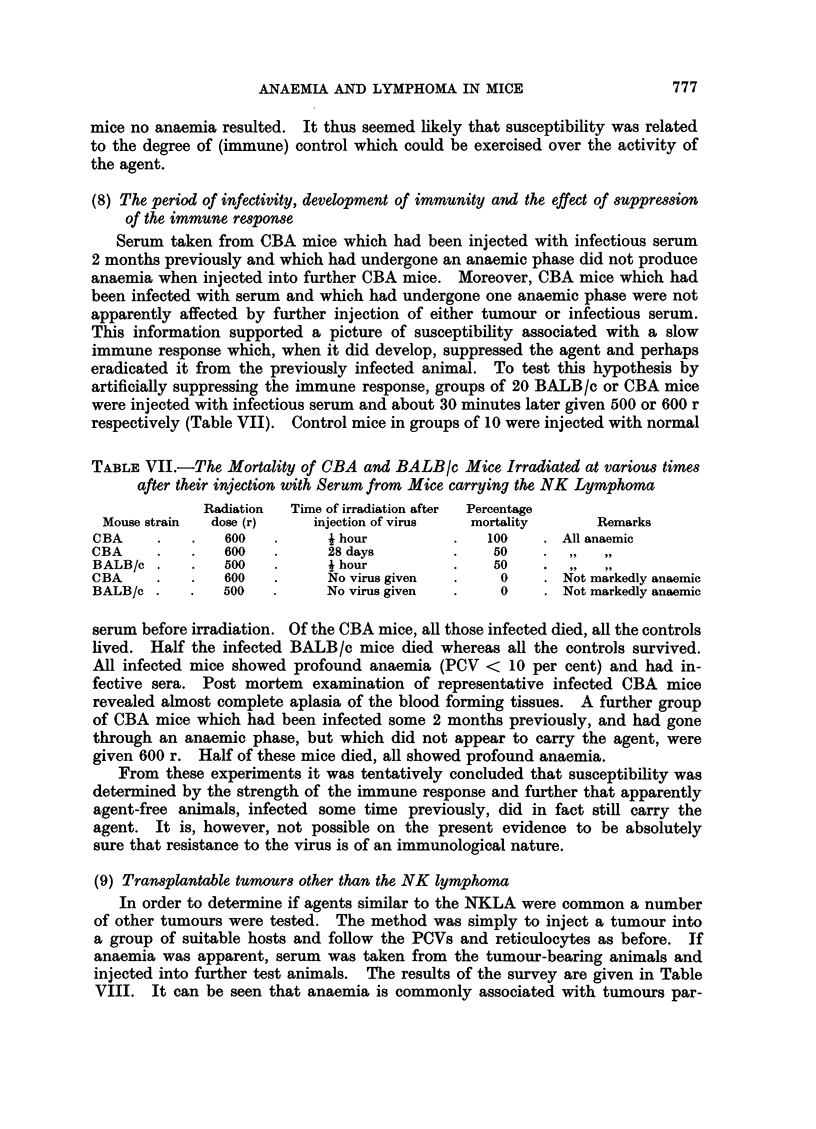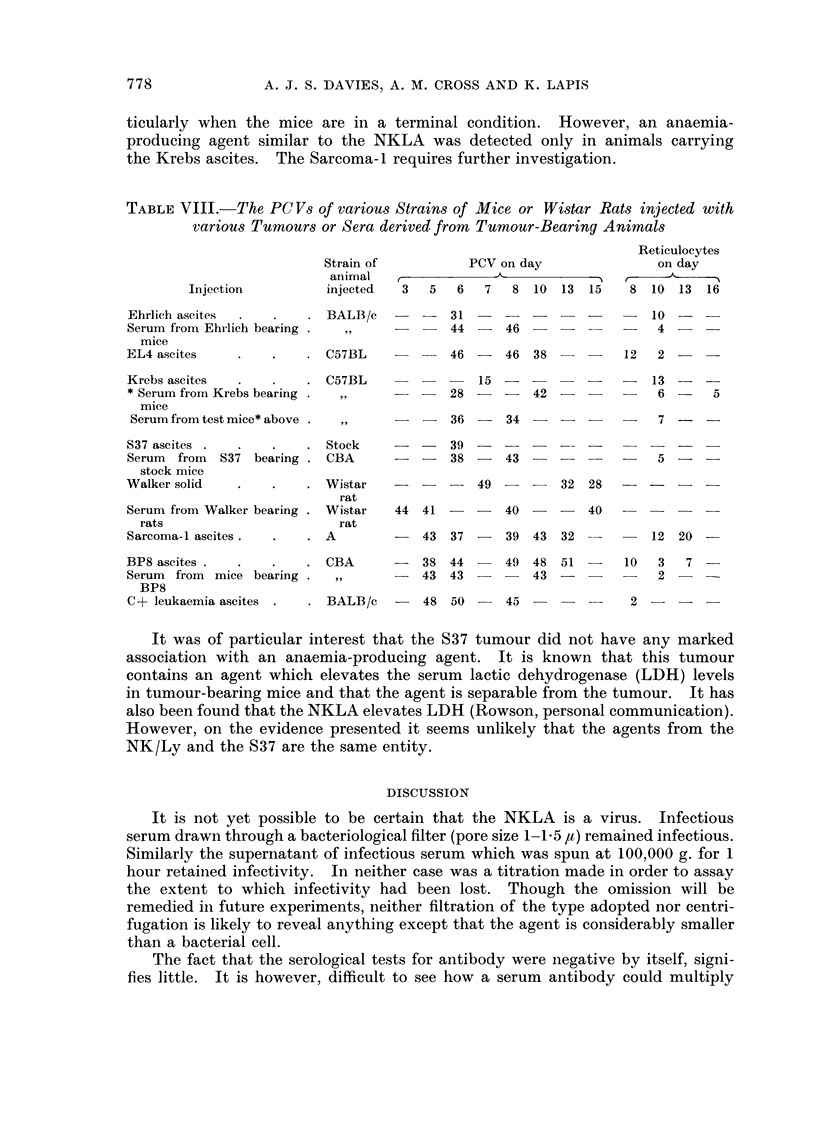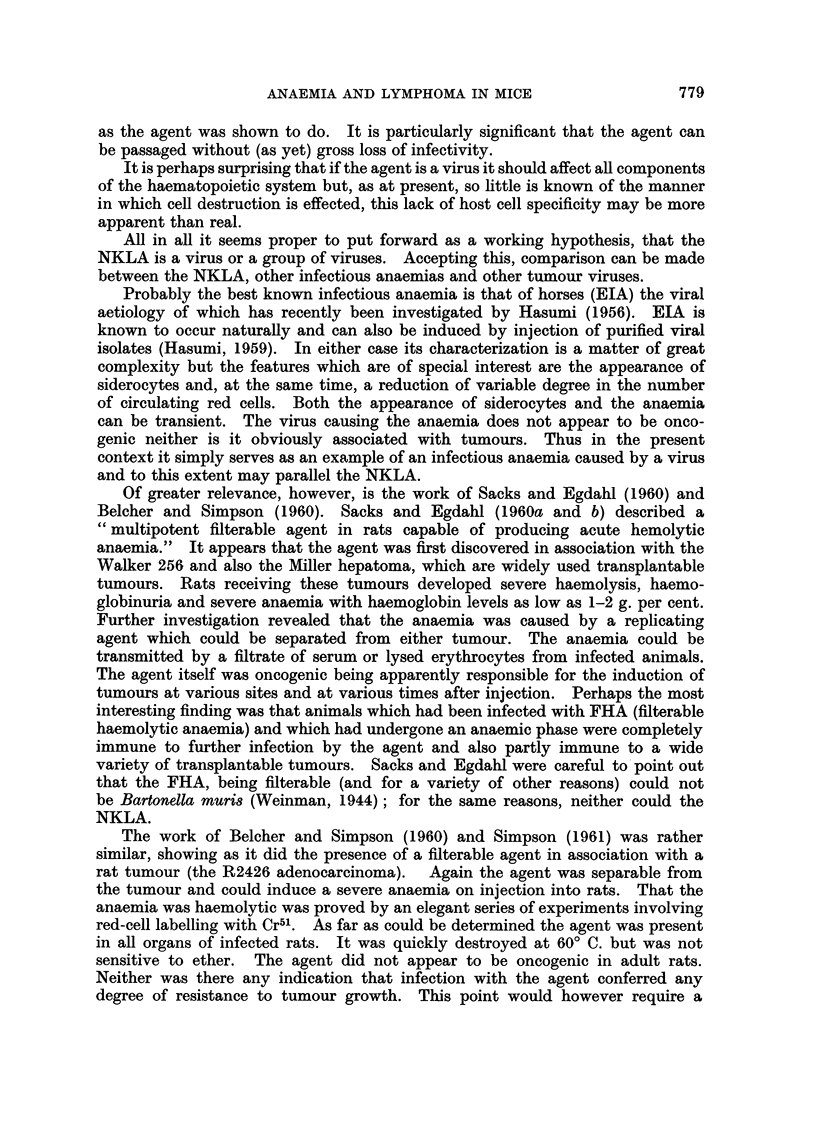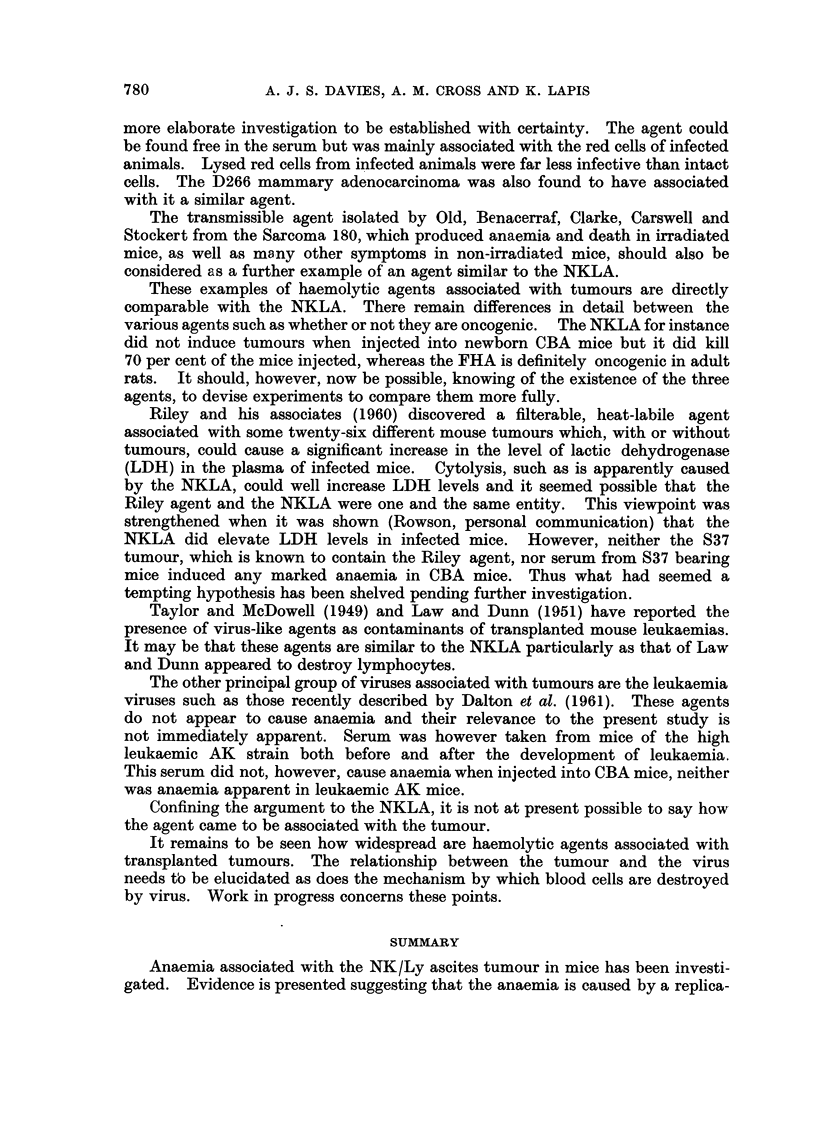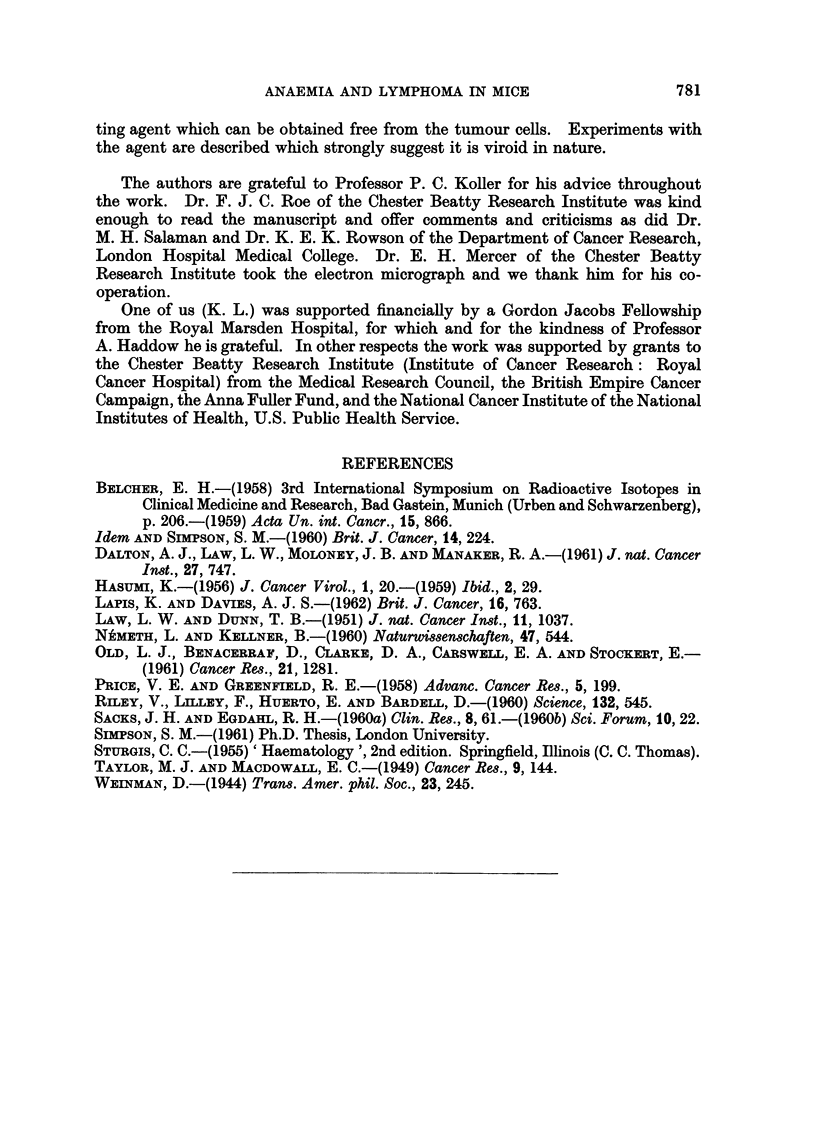# Anaemia Associated with the NK/Lymphoma in Mice

**DOI:** 10.1038/bjc.1962.90

**Published:** 1962-12

**Authors:** A. J. S. Davies, A. M. Cross, K. Lapis

## Abstract

**Images:**


### ANAEMIA ASSOCIATED WITH THE NK/LYMPHOMA IN MICE

A. J. S. DAVIES, A. M. CROSS AND K. LAPIS*

*From the Chester Beatty Research Institute, Institute of Cancer Research, Royal Cancer Hospital, London, S.W.3*

Received for publication August 22, 1962

AN association between anaemia and malignancy is a common finding in man (Sturgis, 1955) and has also been recorded in experimental animals (Price and Greenfield, 1958). In many instances, however, the exact causal relationship between the two conditions is not clear. The available hypotheses, both in general and particular, have been reviewed by Price and Greenfield (1958) and by Simpson (1961). The work of Belcher and Simpson (1960) and of Simpson (1961) is most relevant to the present study. These authors (following preliminary reports by Belcher, 1958, 1959) described red-cell destruction with consequent anaemia in " August " strain rats bearing the transplantable mammary adenocarcinoma, R 2426. It was suggested that the agent responsible, which was separable from the tumour, could be a virus. An investigation of a similar agent (NKLA), associated with the NK/Ly ascites, has been made and will be reported here.

### MATERIALS AND METHODS

*Mice.*—Two to 4 month-old mice of either sex of the CBA strain were used principally. The CBA strain has been maintained by inbreeding in the Chester Beatty Research Institute for many generations and can be considered genetically homogeneous. Other inbred Chester Beatty mouse strains used were the BALB/c, C57BL and A, and colony-bred stock mice. Colony-bred C3 and Swiss mice were used for that part of the work carried out in the Oncopathology Institute, Budapest. The rats used were of the Chester Beatty colony-bred Wistar albino strain.

### *Tumours*

(i) *NK/Ly ascites tumour* (Németh and Kellner, 1960).—The tumour was maintained as ascites in the peritoneal cavity of CBA mice, being passaged about every 10th day. When required for experiment tumour cells were taken 1 week after passage.

(ii) *Other tumours.*—The following transplanted ascites tumours were used: Sa-1, C+ leukaemia, EL4, BP8, Krebs, S37 and Ehrlich (hyperdiploid) growing in the A, BALB/c, C57BL, CBA, C57BL, Stock and BALB/c strains of mice respectively. The last three of these tumours have no notable strain specificities, the remainder may be regarded as strain specific.

* Present address: Research Institute of Oncopathology, Ráth Gyorgy utca 5, Budapest XII, Hungary.

*Methods*

(i) *Haematological.*—Mice were bled from the tail. Estimated components of the blood were haemoglobin (Hb), packed cell volumes (PCV), reticulocytes, platelets, total white blood cells, mononuclear- and polymorphonuclear leucocytes. A micro-haematocrit centrifuge was used for estimation of PCV, otherwise standard haemocytometric methods were employed.

(ii) *Serum and red cells.*—Serum was obtained from blood collected after decapitation. The blood was allowed to stand at 4° C. for at least 1 hour and was then spun at 1700 g. for about 10 minutes. The supernatant was collected and, when necessary, stored at —79° C. in glass ampoules. Serum was injected intravenously into mice in 0·1 ml. aliquots. Red cell suspensions were prepared by diluting whole blood into isotonic citrate : saline (1 : 10). Except where indicated, 0·1 ml. of a 20 per cent red blood cell suspension was injected. No efforts were made to exclude white cells.

### EXPERIMENTS AND THEIR RESULTS

(1) *The basic experiment*

The suspicion that the NK lymphoma was responsible for, or associated with, anaemia in tumour-bearing mice arose from experiments in which attempts were made to arrest the growth of the tumour by means of Degranol (1,6-Di-$\beta$-chloroethylamino-1,6-dideoxy-mannitol) or irradiation (Lapis and Davies, 1962). The basic experiment which confirmed this suspicion was performed by injecting $3 \times 10^6$ cells of NK/Ly ascites into 20 mice and studying their peripheral blood after various time intervals. Five mice were bled at a time, but in order to avoid any effect of bleeding *per se*, no mouse was bled more frequently than once every 8 days.

The results are set out in Fig. 1. It can be seen that both the counts of platelets and white cells were depressed on the 2nd day after injection ($P < 0·01$) but thereafter recovered. The haemoglobin and PCVs fell sharply after the 4th day ($P < 0·01$) but recovered to normal by the 10th day. Associated with the low red cell levels was a considerable reticulocytosis ($P< 0·01$ after the 4th day) commencing about the 4th day and continuing for at least 21 days.

(2) *Anaemia after injection of serum or red cells*

Following Belcher and Simpson (1960) it was decided to determine whether the agent responsible for the anaemia could be obtained free from the tumour. Serum and red cells were taken from tumour-bearing CBA mice and injected, separately, into CBA mice. The PCVs and haemoglobins of the injected mice were recorded on alternate days until apparently stable values were obtained. As before, mice were bled in batches. Both serum and washed red cells caused anaemia and their effects were approximately the same. The recovery to normal haemoglobin levels lagged behind recovery of the PCVs. This is perhaps to be expected if rapid red cell proliferation follows the anaemic phase. In an as yet incomplete series of experiments it has proved possible to passage the agent every 7 days by means of injections of 0·1 ml. of serum from infected mice. Up to the 30th passage no loss of infectivity is apparent.

(3) *Tests for antibody*

It seemed possible that the anaemia observed was due to antibody produced, perhaps by the tumour against the host, perhaps by the host itself, this latter case qualifying as an auto-immune reaction. In either instance it might have been possible to detect antibody in the serum, or coating the red cells, of anaemic

FIG. 1.—The peripheral blood values of CBA mice at various times after their intravenous injection with $3 \times 10^6$ cells of NK/Ly ascites.

| | |
|---|---|
| – · · · · –● · · · · – | reticulocytes |
| – ——●—— – | haemoglobin |
| ——●—— | PCV |
| –O–O ●–O–O | white blood cells |
| – – – –●– – – – | platelets |

mice. Accordingly, a rabbit anti-mouse-serum antiserum was absorbed with mouse red cells, and used in both direct and indirect Coomb's tests. All tests were done at room temperature and at 4° C. All were negative. Neither was it possible to demonstrate that the serum of anaemic mice had any lytic effect *in vitro* upon mouse red cells—various sources of complement having been added to the reaction mixtures. Serum from anaemic mice was absorbed with normal washed red cells (1 vol. serum : 1 vol. packed red cells) twice for 1 hour at 4° C.

This did not abolish the capacity of the serum to produce anaemia upon injection into CBA mice. Though not exhaustive, these tests did not in any way support the hypothesis that an antibody reaction was responsible for the anaemia.

(4) *Assay of NKLA.*

Belcher and Simpson (1960) showed that the apparent amount of agent in tumour-bearing rats was related to the time after tumour implantation. Thus, serum from rats which had received the tumour 1 day previously upon injection into other rats produced no anaemic episode ; serum from rats which had received the tumour 3 days previously, gave a positive reaction within 12 days. Serum taken 14 days after tumour injection elicited an anaemic episode within 2 days. The length of time for the anaemic episode to develop might then be an indication of the amount or activity of the agent present.

An attempt was made to repeat this type of assay. Serum and red cells were taken from CBA mice 2, 4, 6, 8 and 12 days after intravenous injection of $3 \times 10^6$ cells of NK/Ly ascites. The serum and red cells were injected separately into groups of CBA mice and their haemoglobin and PCVs were determined every other day for 12 days. As before, bleeding was done on batches of different mice rather than serially on the same mice. The results are shown in Table I.

TABLE I.—*The PCVs and Haemoglobin Levels (Hb) of CBA Mice at Various Time Intervals after an Injection of Red Cells or Serum from CBA Mice which had at various times previously received* $3 \times 10^6$ *Cells of NK/Ly Ascites*

| Time in days after injection of tumour | Serum (S) or red cells (C) | Time in days after injection of serum or red cells: 2 | | 4 | | 6 | | 8 | | 10 | | 12 | |
|---|---|---|---|---|---|---|---|---|---|---|---|---|---|
| | | PCV | Hb | PCV | Hb | PCV | Hb | PCV | Hb | PCV | Hb | PCV | Hb |
| 2 | C | 54 | 105 | 46 | 87 | 30 | 66 | 34 | 60 | 51 | 108 | 43 | 97 |
| | S | 54 | 109 | 50 | 97 | 29 | 63 | 34 | 62 | 43 | 93 | 46 | 98 |
| 4 | C | 48 | 107 | 49 | 101 | 41 | 100 | 39 | 76 | 44 | 74 | 50 | 106 |
| | S | 49 | 102 | 52 | 100 | 40 | 98 | 44 | 85 | 47 | 81 | 46 | 106 |
| 6 | C | 50 | 95 | 49 | 97 | 34 | 67 | 28 | 56 | 43 | 68 | 46 | 77 |
| | S | 54 | 93 | 39 | 81 | 28 | 57 | 32 | 61 | 46 | 83 | — | 77 |
| 8 | C | 50 | 114 | 38 | 83 | 28 | 53 | 36 | 50 | 42 | 73 | 48 | 83 |
| | S | 52 | 120 | 42 | 80 | 30 | 60 | 40 | 64 | 46 | 78 | 46 | 92 |
| 12 | C | 54 | 101 | 45 | 83 | 30 | 61 | 38 | 63 | 35 | 63 | 51 | 92 |
| | S | 51 | 106 | 34 | 69 | 33 | 74 | 40 | 62 | 45 | 87 | 51 | 92 |

Each value given is a mean from at least three mice. Both sera and red cells appeared to have a similar effect. All injections seemed to have similar potencies. As the PCV and haemoglobin values ran roughly together it was decided to abandon haemoglobin determinations in subsequent investigations. Further, as in every case the PCV was lowest on the 6th or 8th day, it was decided to sample on the 6th day and, if the result was negative, again on the 8th day. In addition reticulocyte determinations were to be made on the 10th day at a time when the maximum degree of reticulocytosis was apparent. As injection of red cells and of sera had given similar results, it was decided to concentrate on injections of sera. On this basis sera were removed and assayed from mice shortly after intravenous injection of the NK lymphoma (Table II). There was a slight indication from the reticulocyte counts that the amount of NKLA declined by 15 hours after

TABLE II.—*The Mean PCV and Reticulocyte Counts of CBA Mice injected with 0·1 ml. of Serum from CBA Mice which had received* 3 × $10^6$ *Cells of the NK/Ly Ascites at various times previously*

| Time in hours after injection of tumour | PCV on day 6 | Reticulocytes per cent on day 10 |
|---|---|---|
| 1 | 30 | 16 |
| 3 | 28 | 21 |
| 6 | 28 | 21 |
| 15 | 32 | 6 |
| 24 | 30 | 7 |

injection of the tumour. It was, however, clear that assay by measuring the time taken to produce anaemia was not useful. Accordingly, it was decided to make various dilutions of sera and red cells from mice 6 days after intravenous injection of NK/Ly ascites, before injection into test mice (Table III).

TABLE III.—*The PCV per cent and Reticulocyte per cent Level of CBA Mice at various times after their Injection with various Dilutions of Serum or Red Cells from Mice which had* 6 *days previously received* 3 × $10^6$ *Cells of NK/Ly Ascites Intravenously*

| Serum or red cells | Dilution | PCV on day 6 | PCV on day 8 | Reticulocytes on day 10 |
|---|---|---|---|---|
| Red cells | 0 | 34 | 28 | Not determined |
| | 1 : 50 | 43 | 28 | 8 |
| | 1 : 500 | 48 | 40 | 5 |
| | 1 : 5,000 | 49 | 46 | 2 |
| | 1 : 500,000 | 46 | 48 | 2 |
| Serum | 0 | 25 | — | 33 |
| | 1 : 100 | 23 | — | 25 |
| | 1 : 1,000 | 42 | 40 | 14 |
| | 1 : 10,000 | 43 | 47 | 4 |
| | 1 : 100,000 | 47 | 45 | 4 |
| | 1 : 1,000,000 | 47 | 46 | 1 |

Without doubt more agent was present in the serum than associated with washed red cells. Further, the method of assay was satisfactory. The dilution study was extended to encompass a wider range of times. Assay was made of agent in the serum of mice which had been injected with serum from tumour-bearing mice. The results are recorded in Table IV; it seemed that the titre of agent free in the serum dropped slightly upon injection, as might be expected with an approximately 1 in 20 dilution (the blood volume of a 20 g. mouse is about 2 ml.). After 15 hours the titre was reduced tenfold and rose 50 fold within the next 33 hours. Six days after injection the titre was lower than at 48 hours, but was thereafter maintained until 14 days. A later experiment, of which the results are shown in Table V, showed clearly that the titre drops sharply within 3 hours of injection and remains low until 12 hours, after which it rises slowly. Such a growth pattern of multiplication might be expected of a virus.

### (5) *Electron microscopy*

Accepting as a working hypothesis that the NKLA was in fact a virus, it was decided to attempt visualization with the electron microscope. Serum

TABLE IV.—*The PCV's, per cent, and reticulocytes, per cent, of CBA mice injected with 0·1 ml. of serum or diluted serum from CBA mice infected at various times previously (first column) with the NKLA.*

| Time after injection of infectious serum | Serum dilution | PCV on day 6 | PCV on day 8 | Reticulocytes per cent on day 10 | Reticulocytes per cent on day 15 |
|---|---|---|---|---|---|
| 1 hour | 0 | 30 | — | 16 | — |
| | 1 : 100 | 29 | — | 22 | — |
| | 1 : 500 | 37 | 38 | 12 | — |
| | 1 : 1,000 | 34 | 33 | 16 | — |
| | 1 : 5,000 | 44 | 40 | 1 | — |
| | 1 : 10,000 | 48 | 47 | 1 | — |
| 15 hours | 0 | 32 | — | 6 | — |
| | 1 : 100 | 34 | 35 | 20 | — |
| | 1 : 500 | 48 | 51 | 3 | — |
| | 1 : 1,000 | 46 | 45 | 4 | — |
| | 1 : 5,000 | 43 | 47 | 4 | — |
| | 1 : 10,000 | 45 | 44 | 3 | — |
| 48 hours | 0 | 29 | — | ? | — |
| | 1 : 100 | 30 | — | 6 | 10 |
| | 1 : 500 | 31 | — | 9 | 8 |
| | 1 : 1,000 | 28 | — | 18 | — |
| | 1 : 5,000 | 26 | — | 14 | — |
| | 1 : 10,000 | 42 | 40 | 2 | — |
| 6 days | 0 | 25 | 34 | 33 | 2 |
| | 1 : 100 | 23 | 39 | 25 | 2 |
| | 1 : 1,000 | 42 | 32 | 14 | 2 |
| | 1 : 10,000 | 43 | 48 | 4 | 2 |
| | 1 : 100,000 | 47 | 52 | 4 | 3 |
| | 1 : 1,000,000 | 47 | 50 | 1 | 3 |
| 14 days | 1 : 100 | 36 | 40 | 7 | — |
| | 1 : 500 | 36 | 38 | 12 | — |
| | 1 : 1,000 | 36 | 39 | 15 | — |
| | 1 : 5,000 | 42 | 42 | 3 | — |
| | 1 : 10,000 | 42 | 43 | 6 | — |

TABLE V.—*The Infective Titres, as measured by ability to induce Anaemia, of Sera of Mice various times after their injection with 0·1 ml. of Serum containing the NK Lymphoma Agent*

| Time in hours after injection | Titre* |
|---|---|
| 0 | >10,000 |
| 3 | <100 |
| 6 | 100 |
| 9 | 100 |
| 12 | 100 |
| 15 | <1,000>100 |
| 18 | 1,000 |
| 21 | 100 |
| 24 | <5,000>1,000 |

* Reciprocal of ultimate infective dilution.

from tumour-bearing and from normal mice was collected and centrifuged at 10,000 g. for 20 minutes to remove large debris. The supernatants were collected and spun at 100,000 g. for 1 hour. The pellets were sectioned and then examined in the electron microscope. The pellet from the serum of tumour-bearing mice contained a number of virus-like particles (Fig. 2) ; that from normal serum did not. The observation of virus-like particles has so far been restricted to two samples of serum from tumour-bearing mice, but it was sufficiently encouraging to continue with an attempt to characterize the postulated virus.

(6) *Heat and ether sensitivity*

Infectious sera were kept for 30 minutes at 37°, 56° or 80° C. preceding injection into test mice. Other sera were treated with ether (1 ml. serum : 0·1 ether) overnight before test. Table VI gives the test results, i.e. virtually complete inactivation at 56° C. and with ether.

TABLE VI.—*The PCVs and Reticulocyte Counts of Mice at various times after injection of Infectious Serum which had been treated in a variety of ways*

| Treatment of serum | PCV on day 6 | PCV on day 8 | Reticulocytes per cent on day 10 |
|---|---|---|---|
| 37°C. for 30 minutes | 22 | — | 20 |
| 56°C. for 30 minutes | 50 | 45 | 2 |
| 80°C. for 30 minutes | 49 | 50 | — |
| Ether for 12 hours | 47 | 45 | 2 |

(7) *Mouse-strain specificity*

All experiments had so far been with CBA mice. A number of experiments were devised to test the specificity of the NKLA. BALB/c and C57Bl mice were injected intravenously with $3 \times 10^6$ cells of the NK/Ly ascites and their PCVs and haemoglobin values followed daily for 15 days. No marked anaemia was observed though the C57Bl showed somewhat depressed haemoglobins (70 per cent normal) on the 10th day after injection of tumour. The PCVs of these mice were not, however, significantly depressed.

Serum was taken from a number of BALB/c mice some 14 days after implantation of tumour, and injected into CBA mice. A slight anaemia resulted. $C_3$ (the strain of origin of the tumour) and a strain of Swiss mice were injected with tumour and components of the blood were analysed for a number of days. The results were equivocal ; some depression of platelets and red cells was observed but the effect was by no means as marked as when the NK/Ly is injected into CBA mice. The inference from all these experiments is either that the agent is specific in its growth requirements or that C3, BALB/c, C57Bl and Swiss mice are not so susceptible to attack as CBA. It was thought possible that the agent was carried but without pathological effect in non-susceptible mice. However, when serum from normal BALB/c mice was injected into the susceptible CBA

EXPLANATION OF PLATE

FIG. 2.—Electron micrograph of a section of the pellet obtained by spinning serum from mice 6 days after the i.p. injection with $3 \times 10^6$ cells of NK/Ly ascites at 100,000 g. for 1 hour. ($\times$80,000.)

2

Davies, Cross and Lapis.

mice no anaemia resulted. It thus seemed likely that susceptibility was related to the degree of (immune) control which could be exercised over the activity of the agent.

(8) *The period of infectivity, development of immunity and the effect of suppression of the immune response*

Serum taken from CBA mice which had been injected with infectious serum 2 months previously and which had undergone an anaemic phase did not produce anaemia when injected into further CBA mice. Moreover, CBA mice which had been infected with serum and which had undergone one anaemic phase were not apparently affected by further injection of either tumour or infectious serum. This information supported a picture of susceptibility associated with a slow immune response which, when it did develop, suppressed the agent and perhaps eradicated it from the previously infected animal. To test this hypothesis by artificially suppressing the immune response, groups of 20 BALB/c or CBA mice were injected with infectious serum and about 30 minutes later given 500 or 600 r respectively (Table VII). Control mice in groups of 10 were injected with normal

TABLE VII.—*The Mortality of CBA and BALB/c Mice Irradiated at various times after their injection with Serum from Mice carrying the NK Lymphoma*

| Mouse strain | Radiation dose (r) | Time of irradiation after injection of virus | Percentage mortality | Remarks |
|---|---|---|---|---|
| CBA | 600 | ½ hour | 100 | All anaemic |
| CBA | 600 | 28 days | 50 | ,, ,, |
| BALB/c | 500 | ½ hour | 50 | ,, ,, |
| CBA | 600 | No virus given | 0 | Not markedly anaemic |
| BALB/c | 500 | No virus given | 0 | Not markedly anaemic |

serum before irradiation. Of the CBA mice, all those infected died, all the controls lived. Half the infected BALB/c mice died whereas all the controls survived. All infected mice showed profound anaemia (PCV $<$ 10 per cent) and had infective sera. Post mortem examination of representative infected CBA mice revealed almost complete aplasia of the blood forming tissues. A further group of CBA mice which had been infected some 2 months previously, and had gone through an anaemic phase, but which did not appear to carry the agent, were given 600 r. Half of these mice died, all showed profound anaemia.

From these experiments it was tentatively concluded that susceptibility was determined by the strength of the immune response and further that apparently agent-free animals, infected some time previously, did in fact still carry the agent. It is, however, not possible on the present evidence to be absolutely sure that resistance to the virus is of an immunological nature.

(9) *Transplantable tumours other than the NK lymphoma*

In order to determine if agents similar to the NKLA were common a number of other tumours were tested. The method was simply to inject a tumour into a group of suitable hosts and follow the PCVs and reticulocytes as before. If anaemia was apparent, serum was taken from the tumour-bearing animals and injected into further test animals. The results of the survey are given in Table VIII. It can be seen that anaemia is commonly associated with tumours particularly when the mice are in a terminal condition. However, an anaemia-producing agent similar to the NKLA was detected only in animals carrying the Krebs ascites. The Sarcoma-1 requires further investigation.

TABLE VIII.—*The PCVs of various Strains of Mice or Wistar Rats injected with various Tumours or Sera derived from Tumour-Bearing Animals*

| Injection | Strain of animal injected | PCV on day | | | | | | | | Reticulocytes on day | | | |
|---|---|---|---|---|---|---|---|---|---|---|---|---|---|
| | | 3 | 5 | 6 | 7 | 8 | 10 | 13 | 15 | 8 | 10 | 13 | 16 |
| Ehrlich ascites | BALB/c | — | — | 31 | — | — | — | — | — | — | 10 | — | — |
| Serum from Ehrlich bearing mice | ,, | — | — | 44 | — | 46 | — | — | — | — | 4 | — | — |
| EL4 ascites | C57BL | — | — | 46 | — | 46 | 38 | — | — | 12 | 2 | — | — |
| Krebs ascites | C57BL | — | — | — | 15 | — | — | — | — | — | 13 | — | — |
| * Serum from Krebs bearing mice | ,, | — | — | 28 | — | — | 42 | — | — | — | 6 | — | 5 |
| Serum from test mice* above | ,, | — | — | 36 | — | 34 | — | — | — | — | 7 | — | — |
| S37 ascites | Stock | — | — | 39 | — | — | — | — | — | — | — | — | — |
| Serum from S37 bearing stock mice | CBA | — | — | 38 | — | 43 | — | — | — | — | 5 | — | — |
| Walker solid | Wistar rat | — | — | — | 49 | — | — | 32 | 28 | — | — | — | — |
| Serum from Walker bearing rats | Wistar rat | 44 | 41 | — | — | 40 | — | — | 40 | — | — | — | — |
| Sarcoma-1 ascites | A | — | 43 | 37 | — | 39 | 43 | 32 | — | — | 12 | 20 | — |
| BP8 ascites | CBA | — | 38 | 44 | — | 49 | 48 | 51 | — | 10 | 3 | 7 | — |
| Serum from mice bearing BP8 | ,, | — | 43 | 43 | — | — | 43 | — | — | — | 2 | — | — |
| C+ leukaemia ascites | BALB/c | — | 48 | 50 | — | 45 | — | — | — | 2 | — | — | — |

It was of particular interest that the S37 tumour did not have any marked association with an anaemia-producing agent. It is known that this tumour contains an agent which elevates the serum lactic dehydrogenase (LDH) levels in tumour-bearing mice and that the agent is separable from the tumour. It has also been found that the NKLA elevates LDH (Rowson, personal communication). However, on the evidence presented it seems unlikely that the agents from the NK/Ly and the S37 are the same entity.

### DISCUSSION

It is not yet possible to be certain that the NKLA is a virus. Infectious serum drawn through a bacteriological filter (pore size 1–1·5 $\mu$) remained infectious. Similarly the supernatant of infectious serum which was spun at 100,000 g. for 1 hour retained infectivity. In neither case was a titration made in order to assay the extent to which infectivity had been lost. Though the omission will be remedied in future experiments, neither filtration of the type adopted nor centrifugation is likely to reveal anything except that the agent is considerably smaller than a bacterial cell.

The fact that the serological tests for antibody were negative by itself, signifies little. It is however, difficult to see how a serum antibody could multiply as the agent was shown to do. It is particularly significant that the agent can be passaged without (as yet) gross loss of infectivity.

It is perhaps surprising that if the agent is a virus it should affect all components of the haematopoietic system but, as at present, so little is known of the manner in which cell destruction is effected, this lack of host cell specificity may be more apparent than real.

All in all it seems proper to put forward as a working hypothesis, that the NKLA is a virus or a group of viruses. Accepting this, comparison can be made between the NKLA, other infectious anaemias and other tumour viruses.

Probably the best known infectious anaemia is that of horses (EIA) the viral aetiology of which has recently been investigated by Hasumi (1956). EIA is known to occur naturally and can also be induced by injection of purified viral isolates (Hasumi, 1959). In either case its characterization is a matter of great complexity but the features which are of special interest are the appearance of siderocytes and, at the same time, a reduction of variable degree in the number of circulating red cells. Both the appearance of siderocytes and the anaemia can be transient. The virus causing the anaemia does not appear to be oncogenic neither is it obviously associated with tumours. Thus in the present context it simply serves as an example of an infectious anaemia caused by a virus and to this extent may parallel the NKLA.

Of greater relevance, however, is the work of Sacks and Egdahl (1960) and Belcher and Simpson (1960). Sacks and Egdahl (1960*a* and *b*) described a " multipotent filterable agent in rats capable of producing acute hemolytic anaemia." It appears that the agent was first discovered in association with the Walker 256 and also the Miller hepatoma, which are widely used transplantable tumours. Rats receiving these tumours developed severe haemolysis, haemoglobinuria and severe anaemia with haemoglobin levels as low as 1–2 g. per cent. Further investigation revealed that the anaemia was caused by a replicating agent which could be separated from either tumour. The anaemia could be transmitted by a filtrate of serum or lysed erythrocytes from infected animals. The agent itself was oncogenic being apparently responsible for the induction of tumours at various sites and at various times after injection. Perhaps the most interesting finding was that animals which had been infected with FHA (filterable haemolytic anaemia) and which had undergone an anaemic phase were completely immune to further infection by the agent and also partly immune to a wide variety of transplantable tumours. Sacks and Egdahl were careful to point out that the FHA, being filterable (and for a variety of other reasons) could not be *Bartonella muris* (Weinman, 1944) ; for the same reasons, neither could the NKLA.

The work of Belcher and Simpson (1960) and Simpson (1961) was rather similar, showing as it did the presence of a filterable agent in association with a rat tumour (the R2426 adenocarcinoma). Again the agent was separable from the tumour and could induce a severe anaemia on injection into rats. That the anaemia was haemolytic was proved by an elegant series of experiments involving red-cell labelling with $Cr^{51}$. As far as could be determined the agent was present in all organs of infected rats. It was quickly destroyed at 60° C. but was not sensitive to ether. The agent did not appear to be oncogenic in adult rats. Neither was there any indication that infection with the agent conferred any degree of resistance to tumour growth. This point would however require a more elaborate investigation to be established with certainty. The agent could be found free in the serum but was mainly associated with the red cells of infected animals. Lysed red cells from infected animals were far less infective than intact cells. The D266 mammary adenocarcinoma was also found to have associated with it a similar agent.

The transmissible agent isolated by Old, Benacerraf, Clarke, Carswell and Stockert from the Sarcoma 180, which produced anaemia and death in irradiated mice, as well as many other symptoms in non-irradiated mice, should also be considered as a further example of an agent similar to the NKLA.

These examples of haemolytic agents associated with tumours are directly comparable with the NKLA. There remain differences in detail between the various agents such as whether or not they are oncogenic. The NKLA for instance did not induce tumours when injected into newborn CBA mice but it did kill 70 per cent of the mice injected, whereas the FHA is definitely oncogenic in adult rats. It should, however, now be possible, knowing of the existence of the three agents, to devise experiments to compare them more fully.

Riley and his associates (1960) discovered a filterable, heat-labile agent associated with some twenty-six different mouse tumours which, with or without tumours, could cause a significant increase in the level of lactic dehydrogenase (LDH) in the plasma of infected mice. Cytolysis, such as is apparently caused by the NKLA, could well increase LDH levels and it seemed possible that the Riley agent and the NKLA were one and the same entity. This viewpoint was strengthened when it was shown (Rowson, personal communication) that the NKLA did elevate LDH levels in infected mice. However, neither the S37 tumour, which is known to contain the Riley agent, nor serum from S37 bearing mice induced any marked anaemia in CBA mice. Thus what had seemed a tempting hypothesis has been shelved pending further investigation.

Taylor and McDowell (1949) and Law and Dunn (1951) have reported the presence of virus-like agents as contaminants of transplanted mouse leukaemias. It may be that these agents are similar to the NKLA particularly as that of Law and Dunn appeared to destroy lymphocytes.

The other principal group of viruses associated with tumours are the leukaemia viruses such as those recently described by Dalton *et al.* (1961). These agents do not appear to cause anaemia and their relevance to the present study is not immediately apparent. Serum was however taken from mice of the high leukaemic AK strain both before and after the development of leukaemia. This serum did not, however, cause anaemia when injected into CBA mice, neither was anaemia apparent in leukaemic AK mice.

Confining the argument to the NKLA, it is not at present possible to say how the agent came to be associated with the tumour.

It remains to be seen how widespread are haemolytic agents associated with transplanted tumours. The relationship between the tumour and the virus needs to be elucidated as does the mechanism by which blood cells are destroyed by virus. Work in progress concerns these points.

### SUMMARY

Anaemia associated with the NK/Ly ascites tumour in mice has been investigated. Evidence is presented suggesting that the anaemia is caused by a replicating agent which can be obtained free from the tumour cells. Experiments with the agent are described which strongly suggest it is viroid in nature.

The authors are grateful to Professor P. C. Koller for his advice throughout the work. Dr. F. J. C. Roe of the Chester Beatty Research Institute was kind enough to read the manuscript and offer comments and criticisms as did Dr. M. H. Salaman and Dr. K. E. K. Rowson of the Department of Cancer Research, London Hospital Medical College. Dr. E. H. Mercer of the Chester Beatty Research Institute took the electron micrograph and we thank him for his co-operation.

One of us (K. L.) was supported financially by a Gordon Jacobs Fellowship from the Royal Marsden Hospital, for which and for the kindness of Professor A. Haddow he is grateful. In other respects the work was supported by grants to the Chester Beatty Research Institute (Institute of Cancer Research: Royal Cancer Hospital) from the Medical Research Council, the British Empire Cancer Campaign, the Anna Fuller Fund, and the National Cancer Institute of the National Institutes of Health, U.S. Public Health Service.

### REFERENCES

BELCHER, E. H.—(1958) 3rd International Symposium on Radioactive Isotopes in Clinical Medicine and Research, Bad Gastein, Munich (Urben and Schwarzenberg), p. 206.—(1959) *Acta Un. int. Cancr.*, **15**, 866.

*Idem* AND SIMPSON, S. M.—(1960) *Brit. J. Cancer*, **14**, 224.

DALTON, A. J., LAW, L. W., MOLONEY, J. B. AND MANAKER, R. A.—(1961) *J. nat. Cancer Inst.*, **27**, 747.

HASUMI, K.—(1956) *J. Cancer Virol.*, **1**, 20.—(1959) *Ibid.*, **2**, 29.

LAPIS, K. AND DAVIES, A. J. S.—(1962) *Brit. J. Cancer*, **16**, 763.

LAW, L. W. AND DUNN, T. B.—(1951) *J. nat. Cancer Inst.*, **11**, 1037.

NÉMETH, L. AND KELLNER, B.—(1960) *Naturwissenschaften*, **47**, 544.

OLD, L. J., BENACERRAF, D., CLARKE, D. A., CARSWELL, E. A. AND STOCKERT, E.—(1961) *Cancer Res.*, **21**, 1281.

PRICE, V. E. AND GREENFIELD, R. E.—(1958) *Advanc. Cancer Res.*, **5**, 199.

RILEY, V., LILLEY, F., HUERTO, E. AND BARDELL, D.—(1960) *Science*, **132**, 545.

SACKS, J. H. AND EGDAHL, R. H.—(1960*a*) *Clin. Res.*, **8**, 61.—(1960*b*) *Sci. Forum*, **10**, 22.

SIMPSON, S. M.—(1961) Ph.D. Thesis, London University.

STURGIS, C. C.—(1955) ‘ Haematology ’, 2nd edition. Springfield, Illinois (C. C. Thomas).

TAYLOR, M. J. AND MACDOWALL, E. C.—(1949) *Cancer Res.*, **9**, 144.

WEINMAN, D.—(1944) *Trans. Amer. phil. Soc.*, **23**, 245.